# Cabozantinib targets bone microenvironment modulating human osteoclast and osteoblast functions

**DOI:** 10.18632/oncotarget.15390

**Published:** 2017-02-16

**Authors:** Marco Fioramonti, Daniele Santini, Michele Iuliani, Giulia Ribelli, Paolo Manca, Nicola Papapietro, Filippo Spiezia, Bruno Vincenzi, Vincenzo Denaro, Antonio Russo, Giuseppe Tonini, Francesco Pantano

**Affiliations:** ^1^ Department of Medical Oncology, Campus Bio-Medico University of Rome, Rome, Italy; ^2^ Department of Orthopaedics and Trauma Surgery, University Campus Bio-Medico of Rome, Rome, Italy; ^3^ Department of Surgical, Oncological and Oral Sciences, Section of Medical Oncology, University of Palermo, Palermo, Italy

**Keywords:** cabozantinib, bone microenvironment, receptor activator of nuclear factor-kb ligand (RANKL), osteoprotegerin (OPG), human primary cells

## Abstract

Cabozantinib, a c-MET and vascular endothelial growth factor receptor 2 inhibitor, demonstrated to prolong progression free survival and improve skeletal disease-related endpoints in castration-resistant prostate cancer and in metastatic renal carcinoma. Our purpose is to investigate the direct effect of cabozantinib on bone microenvironment using a total human model of primary osteoclasts and osteoblasts.

Osteoclasts were differentiated from monocytes isolated from healthy donors; osteoblasts were derived from human mesenchymal stem cells obtained from bone fragments of orthopedic surgery patients. Osteoclast activity was evaluated by tartrate resistant acid phosphatase (TRAP) staining and bone resorption assays and osteoblast differentiation was detected by alkaline phosphatase and alizarin red staining.

Our results show that non-cytotoxic doses of cabozantinib significantly inhibit osteoclast differentiation (p=0.0145) and bone resorption activity (p=0.0252). Moreover, cabozantinib down-modulates the expression of osteoclast marker genes, *TRAP* (p=0.006), *CATHEPSIN K* (p=0.004) and *Receptor Activator of Nuclear Factor k B* (*RANK*) (p=0.001). Cabozantinib treatment has no effect on osteoblast viability or differentiation, but increases osteoprotegerin mRNA (p=0.015) and protein levels (p=0.004) and down-modulates *Receptor Activator of Nuclear Factor k B Ligand* (*RANKL*) at both mRNA (p<0.001) and protein levels (p=0.043). Direct cell-to-cell contact between cabozantinib pre-treated osteoblasts and untreated osteoclasts confirmed the indirect anti-resorptive effect of cabozantinib.

We demonstrate that cabozantinib inhibits osteoclast functions “directly” and “indirectly” reducing the RANKL/osteoprotegerin ratio in osteoblasts.

## INTRODUCTION

Cabozantinib is an orally bioavailable receptor tyrosine kinase inhibitor with a strong activity against c-MET and vascular endothelial growth factor receptor 2 (VEGFR2) that promote tumour progression and angiogenesis. Osteoclasts and osteoblasts also express c-MET and VEGFR2 [1–4] and secrete the only known ligand for c-MET, the hepatocyte growth factor (HGF), supporting the importance of the HGF/MET signaling axis in the regulation of bone remodelling. [5–8].

Pre-clinical studies in animal models of prostate cancer bone metastases showed that cabozantinib inhibits tumour proliferation and bone resorption, indicating that both the tumour and bone microenvironment may represent cabozantinib targets [9–11]. Moreover, in phase II studies of castration resistant prostate cancer (CRPC) patients, cabozantinib was associated with an increased resolution in bone scans, a pain relief in more than 60% of patients and a marked improvement in progression free survival (PFS) compared with placebo [12–14]. In the subsequent phase III trial (COMET-1), cabozantinib did not increase the overall survival (OS) (primary end-point) compared to prednisone. However, cabozantinib was associated with an improvement in bone scan responses at week 12 (42% for cabozantinib versus 3% for prednisone), in progression-free survival (median of 5.5 months in cabozantinib group vs 2.8 months in prednisone group) and with a reduction of skeletal related event (SRE) rates (14% among patients on cabozantinib and 21% in patients on prednisone) [15]. Recently, a phase III study (METEOR) showed that cabozantinib reduced the risk of disease progression or death compared to everolimus in patients with metastatic renal cell carcinoma (RCC) [16]. Furthermore, in a pre-specified analysis in the subgroup of patients with metastatic bone disease treated with cabozantinib (23%), a marked prolongation of PFS was observed (7.4 months in the cabozantinib arm vs 2.7 months in the everolimus arm). Moreover, SRE, in men who showed previous events, were observed in 15 of 91 patients (16%) in the cabozantinib arm and in 31 of 90 patients (34%) in the everolimus arm [17, 18].

Overall, these clinical and preclinical data support the hypothesis that bone microenvironment may represent a potential mediator of observed treatment responses, but it is not yet clear if cabozantinib acts on bone metastases directly or indirectly regulating osteoclasts and osteoblasts, or both.

Previous works explored the biological activity of cabozantinib on bone tissue using only murine models or established cell lines [19, 20]. Cell lines are helpful at the early phases of evaluating the therapeutic drugs, but they do not fully reproduce the physiology of primary cells and species-related differences could represent a drawback for the translation of the outcomes from bench to bedside [21, 22].

This is the first study investigating cabozantinib effect on bone microenvironment using a total human primary model.

## RESULTS

### c-MET and VEGFR2 are expressed during osteoclast and osteoblast differentiation

We evaluated *c-MET* and *VEGFR2* levels at different stages of osteoclast/osteoblast differentiation by real-time PCR. In particular *c-MET* and *VEGFR2* gene expression was assessed at three different time-points in osteoclasts and osteoblasts [day 0 (monocytes), day 6 (pre- osteoclasts), day 12 (mature osteoclasts); day 0 (mesenchymal cells), day 14 (pre- osteoblasts) and day 21 (mature osteoblasts) respectively].

We found that *c-MET* mRNA levels were significantly increased in the early stages of osteoclast differentiation (monocytes vs pre- osteoclasts p < 0.001; monocytes vs osteoclasts p <0.001), while *VEGFR2* was more expressed in mature osteoclasts (monocytes vs pre- osteoclasts p < 0.001; monocytes vs osteoclasts p < 0.001) (Supplementary Figure 1A). Both *c-MET* and *VEGFR2* mRNA levels remained unchanged during the osteoblast maturation process (Supplementary Figure 1B).

C-MET protein expression decreased during osteoclast differentiation (Supplementary Figure 1C), but was stable during osteoblast maturation (Supplementary Figure 1D). VEGFR2 protein levels were detected in neither osteoblasts nor osteoclasts by western blot analysis.

### Cabozantinib decreases primary osteoclast differentiation and activity

Cabozantinib was administered to osteoclasts at a non-cytotoxic concentration (0,3 μM) (Supplementary Figure 2), every 3 days (from day 0 to day 12) according to other preclinical studies on murine models [11, 19, 20].

**Figure 1:**
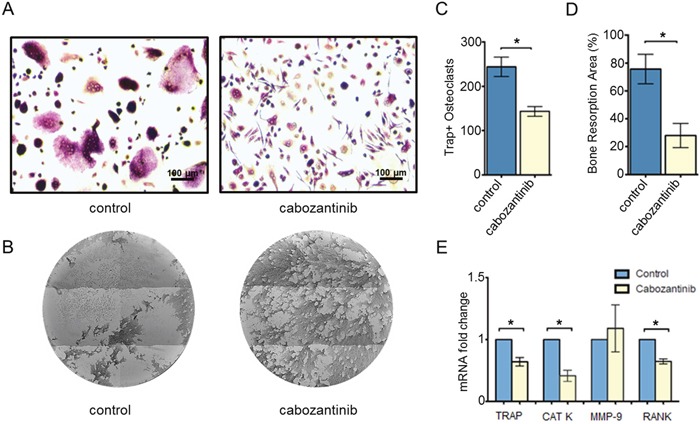
Effect of cabozantinib treatment on primary osteoclasts **A**. Representative images of Trap Assay in untreated or treated osteoclasts (DMSO). **B**. Representative images of Osteoassay in untreated or treated osteoclast (DMSO) which show bone matrix (dark color) and resorption pits (light color) **C**. Count of TRAP+ osteoclasts (≥ 3 nuclei) and **D**. quantification of bone resorption area in untreated or treated osteoclasts. **E**. Modulation of *TRAP*, *CAT K*, *MMP-9* and *RANK* expression levels (Real Time PCR) following cabozantinib treatment *(p < 0.05)

Data showed that cabozantinib treatment had a significant inhibitory effect on osteoclast differentiation reducing the number of tartrate resistant acid phosphatase (TRAP) positive osteoclasts compared to control (DMSO) *(p = 0.0145)* (Figure 1A–1B).

We assessed the effect of cabozantinib treatment on osteoclast activity by seeding monocytes on wells coated with inorganic calcium phosphate that mimic bone matrix and, then, evaluating the reabsorbed areas (pits) produced by mature osteoclasts. We found that cabozantinib significantly inhibited osteoclast function reducing their capacity to reabsorb bone matrix (p = 0.0252) (Figure 1C).

### Cabozantinib does not influence primary osteoblast differentiation and activity

**Figure 2:**
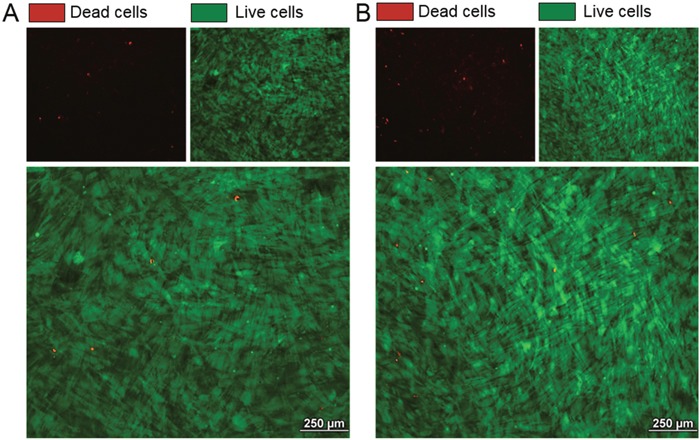
Effect of cabozantinib treatment on osteoblast viability Representative images of Live/Dead staining in untreated osteoblasts **A**. and osteoblasts treated with cabozantinib **B**. Live cells (green), Dead cells (red)

**Figure 3:**
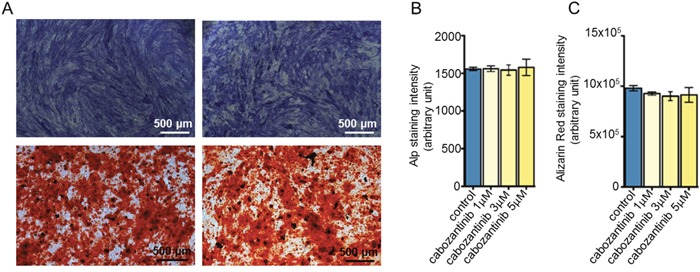
Effect of cabozantinib treatment on primary osteoblast **A**. Representative images of Alp and Alizarin Red assay in untreated or treated osteoblasts (DMSO). **B**. Alp staining and C. Alizarin Red fluorescence quantification in untreated or treated osteoblasts (DMSO).

The effect of cabozantinib on osteoblasts was evaluated via treating the cells from day 21 to day 28 of the differentiation protocol at different concentrations (1 μM, 3 μM and 5 μM). Live and Dead analyses showed that cabozantinib treatment has no cytotoxic effects at these doses (Figure 2); data was confirmed by MTT assay (data not shown). No significant differences in Alkaline Phosphatase (ALP) intensity, a specific osteoblasts marker (Figure 3A-3B) or in Alizarin red (Figure 3C; Supplementary Figure 3), that stains bone matrix deposits, were found suggesting that cabozantinib did not inhibit the ability of primary human osteoblasts to differentiate and mineralize.

### Cabozantinib modulates expression of osteoclast/osteoblast marker genes

Gene expression analysis showed that cabozantinib significantly down-modulated osteoclast markers such as *Tartrate Resistant Acid Phosphatase (TRAP) (p = 0.006)*, *CATHEPSIN K (p= 0.004)* and *Receptor Activator of Nuclear Factor (RANK) (p = 0.001)* but it did not affect *METALLOPROTEINASE-9* (*MMP-9*) expression, a type IV collagenase highly expressed in activated osteoclasts [23] (Figure 1D).

Although we did not find a significant increase of osteoblast mineralization following cabozantinib treatment, molecular analysis revealed a significant up-regulation of some osteoblastogenesis markers such as *Runt-related transcription factor 2* (*RUNX2*) (p = 0.003) and *OSTERIX* (p = 0.015), a down-modulation of *OSTEOCALCIN* (*OCN*), while *ALP* mRNA levels did not change in treated osteoclasts.

**Figure 4:**
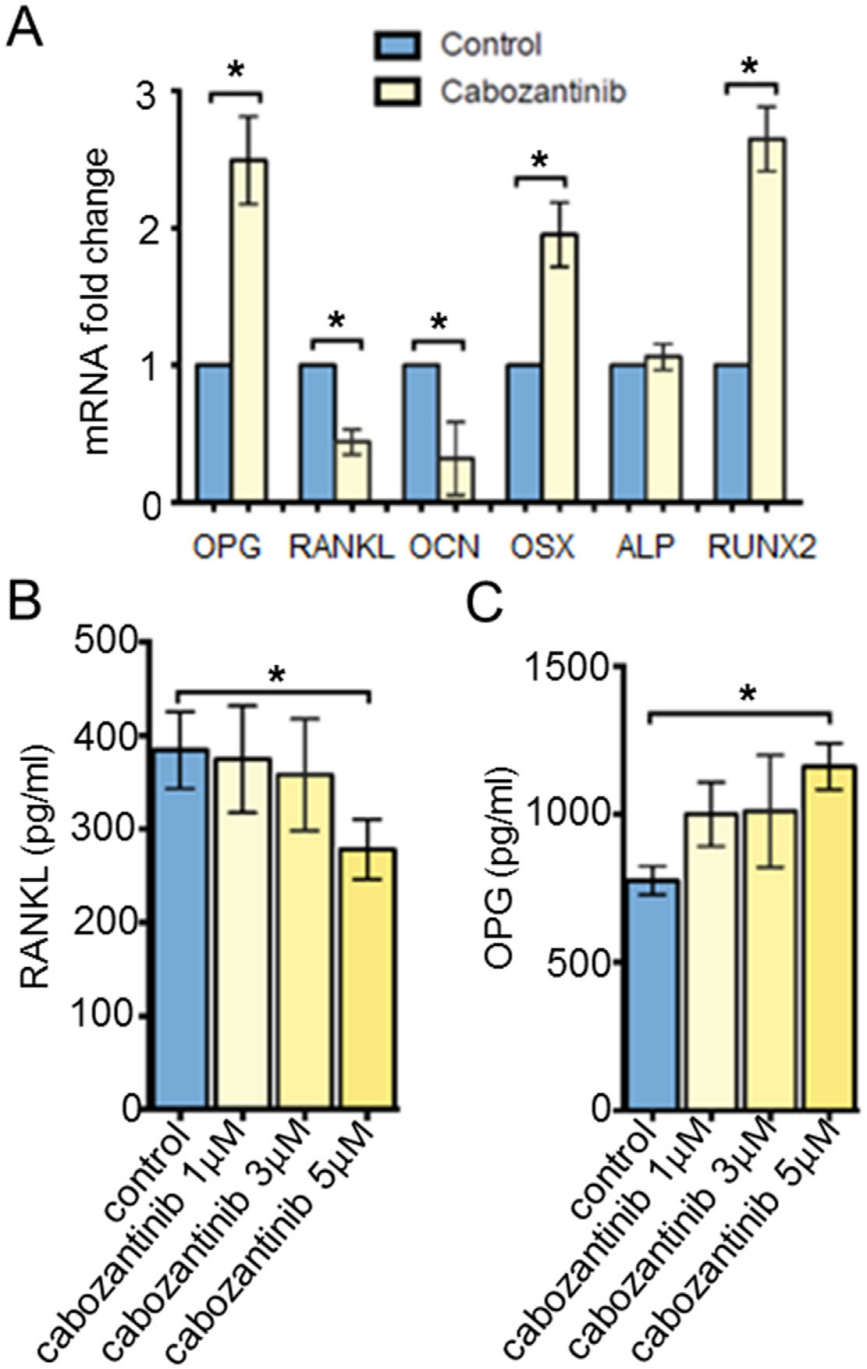
Gene and protein expression analyses **A**. *OPG, RANKL, OCN, OSX, ALP* and *RUNX2* mRNA levels (RealTime PCR) in untreated or treated osteoblasts (DMSO). **B**. RANKL and **C**. OPG protein secretion (ELISA) in untreated or treated osteoblasts. *(p < 0.05)

Receptor activator of nuclear factor-kB ligand (RANKL) binds RANK, promoting osteoclast differentiation and activity. Osteoprotegerin (OPG) is a decoy receptor that blocks RANKL and prevents RANK pathway activation. Balance between RANKL/OPG is crucial for bone resorption regulation [24]. We observed that cabozantinib affected RANKL/OPG ratio upregulating *OPG* mRNA levels *(p = 0.015)* and decreasing *RANKL* expression *(p < 0.001)* (Figure 4A).

### Cabozantinib modulates osteoblast-related protein secretion of soluble OPG and RANKL

In order to confirm the role of cabozantinib on RANKL/OPG axis, we detected the amount of OPG and RANKL proteins secreted in the conditioned media (CM) of untreated or treated osteoblasts. We found that cabozantinib treatment (5 μM) inhibited RANKL secretion *(p = 0.043)* and increased OPG production *(p = 0.004)* by osteoblasts (Figure 4B–4C) [29].

### Osteoblasts pre- treated with cabozantinib exert an inhibitory effect on osteoclast differentiation

**Figure 5:**
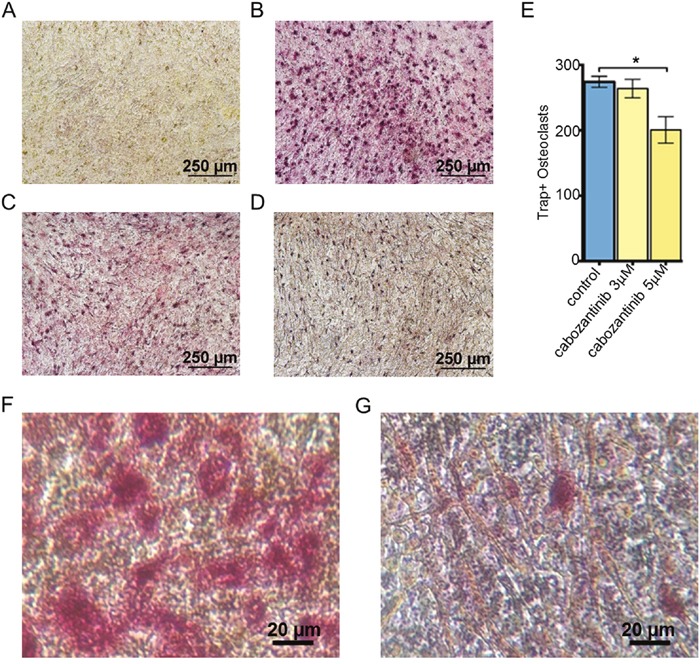
Effect of cabozantinib on osteoclast formation on cabozantinib pre-treated osteoblast layer TRAP-positive cells (red) identify osteoclasts. Representative images: **A**. Osteoblasts cultured without osteoclasts; **B**. Osteoblasts co-cultured with osteoclasts; **C**. Osteoblasts pre-treated with cabozantinib (3 μM) and co-cultured with osteoclasts; **D**. Osteoblasts pre-treated with cabozantinib (5 μM) and co-cultured with osteoclasts; **E**. Number of TRAP-positive cells counted in the three different conditions (B,C and D). F and G. Higher magnification of pictures B and D respectively

We set up an osteoblast/ osteoclast direct co-culture to investigate if osteoblasts pre-treated with cabozantinib exerted an inhibitory effect on osteoclast differentiation. The results showed that the number of TRAP–positive cells (osteoclasts) were significantly reduced in cabozantinib (5 μM) pre-treated osteoblast compared to untreated osteoblasts (Figure 5).

## DISCUSSION

Starting from preclinical and clinical evidences, we designed an *in vitro*/translational study in order to investigate the biological effect of cabozantinib in our models of primary human osteoblasts/osteoclasts. Our results highlight that cabozantinib exerts a strong inhibitory action on osteoclast differentiation and bone-resorbing activity without generating any cytotoxic effect at the administrated doses. In addition, cabozantinib is able to down-modulate mRNA expression of key osteoclastic genes such as *TRAP*, *CATHEPSIN K* and *RANK*.

**Figure 6:**
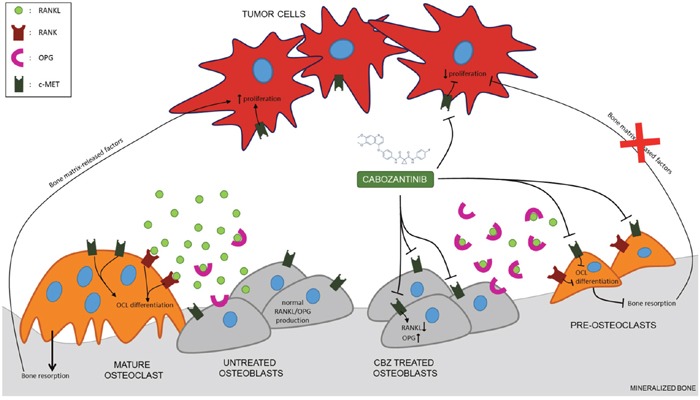
Proposed mechanism of action of cabozantinib in tumor bone microenvironment Cabozantinib could act at multiple levels in the bone metastatic process, targeting cancer cells, bone cells and interfering with the crosstalk between tumour and bone microenvironment through the modulation of RANKL/OPG axis.

Cabozantinib does not inhibit osteoblast differentiation and bone matrix deposition, but, most interestingly, we observed a down-modulation of *RANKL* concomitantly with an upregulation of *OPG* levels following the treatment. Thus, we set up an osteoblasts/osteoclasts co-culture (cell-to-cell contact) to evaluate the effect of cabozantinib in a more physiological system. Our results show that cabozantinib pre-treated osteoblasts are able to reduce the number of osteoclasts differentiated directly on the osteoblast layer. Therefore, cabozantinib could act at multiple levels in the bone metastatic process, targeting cancer cells [10–13], bone cells and interfering with the crosstalk between tumour and bone microenvironment. In particular, cabozantinib treatment could interrupt the ‘vicious cycle’ of pro-tumour interactions between osteoblasts, osteoclasts and tumour cells inhibiting bone resorption and the release of bone matrix embedded factors that in turn stimulate tumour cells growth (Figure 6). Moreover, given the well-known role of the RANKL/OPG/RANK axis in bone metastases formation [24], cabozantinib could inhibit the cancer cells homing to bone niche and their subsequent proliferation.

Our *in vitro* results provide the evidence of potential effects of Cabozantinib on both osteoblasts and osteoclasts as components of its *in vivo* efficacy in reducing tumour burden, prolonging skeletal PFS, decreasing new bone metastases onset and delaying the appearance of SREs as demonstrated in metastatic prostate cancer patients [12–14] and more recently, in advanced renal carcinoma [16].

From a clinical perspective, these results could represent the biological rationale to design novel clinical trials in order to investigate Cabozantinib'*s* activity on skeletal disease-related endpoints and its potential synergism with standard antiresorptive agents [25–26] in patients with bone metastatic solid tumors.

## MATERIALS AND METHODS

### Human osteoclasts

Primary human osteoclasts were differentiated from human peripheral blood mononuclear cells (PBMCs) of ten male healthy blood donors following signed informed consent as previously described [27]. The procedure was approved by Ethical Committee of Campus Bio-Medico University of Rome (Prot 21/15 OSS) in accordance with the Declaration of Helsinki principles. Briefly, CD14^+^ monocytes were differentiated in osteoclasts in presence of 25 ng/mL macrophage-colony stimulating factor (M-CSF) and 50 ng/mL receptor activator of Nuclear Factor kappa-B ligand (RANKL) (R&D Systems). Cabozantinib (0,3 μM) was added every 3 days to the culture medium starting from day 0 to the end of the differentiation protocol (day 12).

### Human osteoblasts

Primary human osteoblasts were differentiated from human mesenchymal stem cells (hMSCs). Bone fragments of non-oncological orthopedic surgery patients were collected following the approval by Ethical Committee of Campus Bio-Medico University of Rome (Prot 21/15 OSS) in accordance with the Declaration of Helsinki principles. The informed consent was obtained from all subjects. Bone fragments were digested with Collagenase II (1.5 mg/mL)/ Tripsin (1X) mix solution for 2 hours at 37°C and placed in a culture flask with alpha MEM (Euroclone) supplemented with 20% fetal bovine serum (Hyclone, Thermo Scientific), 100 units/ml penicillin, 100 mg/ml streptomycin and amphotericin B (250ng/mL) (basal medium) in order to allow HMSCs migration to the flask surface. osteoblast differentiation was achieved by culturing hMSCs in basal medium supplemented with 10 mM beta-glycerophosphate (Sigma-Aldrich), 50 μM ascorbic acid (Sigma-Aldrich) and 100 nM dexamethasone. Cabozantinib was administrated to mature osteoblasts from day 21 to day 28 (every three days) at different concentrations (1 μM, 3 μM and 5 μM).

### Osteoclast functional assays

At the end of the osteoclasts differentiation protocol (day 12), culture medium was removed and cells were fixed with 4% formaldehyde for 5 minute and stained with leukocyte acid phosphatase (TRAP) kit (Sigma-Aldrich) according to the manufacturer's instructions. Stained positive cells (>3 nuclei) were then counted. Osteoclast activity was assessed by culturing cells on plates coated with a synthetic inorganic bone mimetic matrix (Osteoassay, Corning). At day 12 the culture medium was removed and plates filled with sodium hypochlorite solution to evaluate the ability of mature osteoclasts to reabsorb this substrate; the pits produced by osteoclasts were quantified by ImageJ software [27].

### Osteoblast functional assays

On day 21, cells were fixed with 4% formaldehyde for 5 minutes and stained with an alkaline phosphatase (ALP) kit (Sigma-Aldrich) according to the manufacturer's protocol. ALP positivity was quantified by ImageJ software. In order to detect bone matrix deposition as a marker of osteoblastic activity, cells were fixed with 4% formaldehyde for 20 minute and stained with alizarin red for 1 hour at room temperature. Alizarin red fluorescence was detected at 470 nm and quantified by spectrofluorimeter (Tecan Infinite M200Pro).

### Cell viability

Cell viability was performed on osteoblasts treated with cabozantinib using LIVE/DEAD Viability/Cytoxicity Kit (Thermo Fisher Scientific) according to the manufacturer's protocol.

### Osteoblast-osteoclast co-culture

In order to evaluate the “indirect” effect of cabozantinib on osteoclasts differentiation, we seeded monocytes directly on osteoblasts untreated or previously treated with cabozantinib. osteoblasts/osteoclasts co-culture was performed in basal medium supplemented with Vit D (10^−7^ M) adding neither cabozantinib nor others pro- osteoblasts/osteoclasts cytokines. At day 12, osteoclasts were detected on osteoblasts layer using TRAP staining kit (Sigma-Aldrich) and counted.

### RNA extraction and gene expression analysis

Total RNA was extracted from osteoclasts and osteoblasts at the end of the differentiation protocol using the Trizol reagent (Invitrogen) according to the manufacturer's instructions. RNA was treated with DNase buffer and DNase (DNAse Turbo, Applied Biosystems) to avoid genomic DNA contamination. cDNA was produced using the High Capacity cDNA Reverse Transcription kit (Applied Biosystems) according to the manufacturer's instructions. mRNA levels were measured by quantitative real-time polymerase chain reaction (qRT-PCR) using TaqMan Gene Expression Assays in 7900HT Real-Time PCR System (Applied Biosystems). *c-MET* (Hs01565584_m1), *Vascular Endothelial Growth Factor Receptor 2*, (*VEGFR2*) (Hs00911700_m1), *Tartrate Resistant Acid Phosphatase* (*TRAP*) (Hs00356261_m1), *Cathepsin-K* (*CAT K*) (Hs00166156_m1), *Metalloproteinase-9*, (*MMP-9*) (Hs00234579_m1), *Receptor Activator of Nuclear Factor (NF)-κB* (*RANK*) (Hs00921372_m1), *Alkaline Phosphatase* (*ALP*) (Hs01029144_m1), *Osteocalcin* (*OCN*) (Hs00234160_m1), *Receptor Activator of Nuclear Factor (NF)-κB Ligand* (*RANKL*) (Hs00243522_m1), *Osteoprotegerin* (*OPG*), (Hs00900358_m1) and *Runt-related transcription factor 2* (*RUNX2*) (Hs00231692_m1) expression levels were normalized to the endogenous housekeeping gene *Glucuronidase Beta* (*GUSβ*) (Hs99999908_m1) in both untreated and treated samples using the ΔCT calculation. Subsequently the relative expression levels in treated samples were normalized to the mRNA levels detected in control samples using the ΔΔCT calculation [28].

### Protein extraction and western blot analysis

Cell lysates were obtained using radioimmunoprecipitation assay buffer (RIPA buffer) (Sigma-Aldrich) and quantified using DC protein assay kit (Bio-Rad). Total protein extract (20 mg) from each sample was loaded on 10% SDS-PAGE gels, transferred onto nitrocellulose membranes through the Trans- Blot Turbo Transfer System (Bio-Rad) and incubated in a blocking buffer (TBST 1X with 5% non-fat dry milk) for one hour. Rabbit monoclonal anti-human c-MET (8198S - Cell Signalling), rabbit monoclonal anti-human VEGFR2 (55B11 - Cell Signalling) and mouse anti-human Actin-β (A1978 - Sigma-Aldrich) were incubated for 2 hours at room temperature. Anti-rabbit/mouse HRT-coniugated antibody (Abcam) was used and the chemiluminescence signal detected using ChemiDoc (Bio-Rad) and Quantity One software (Bio-Rad) to quantify the bands’ signal intensity.

### Soluble OPG and RANKL protein secretion assay

Osteoblasts conditioned media (CM) of both treated with cabozantinib or untreated were collected to evaluate the soluble OPG and RANKL protein secretion levels using Human Osteoprotegerin ELISA Kit (RayBio®) and the Total sRANKL ELISA kit (ImmunDiagnostik). Then cells were harvested and counted. RANKL and OPG levels were normalized to cell numbers in each well.

### Statistical analysis

Data were analyzed using the Student t test and One-Way ANOVA test followed by Tukey's multiple comparison tests. The graphics processing and statistical tests were performed using the program GraphPad Prism (San Diego, CA).

## SUPPLEMENTARY MATERIALS FIGURES


