# Supplementary material for: Cabozantinib targets bone microenvironment modulating human osteoclast and osteoblast functions
Source: Oncotarget. 2017 Feb 16;8(12):20113–21. doi: 10.18632/oncotarget.15390 (PMC5386748; doi:10.18632/oncotarget.15390)
Supplement: Supplementary file 1 [file oncotarget-08-20113-s001.pdf]

Cabozantinib targets bone microenvironment modulating human osteoclast and osteoblast functions

SUPPLEMENTARY FIGURES

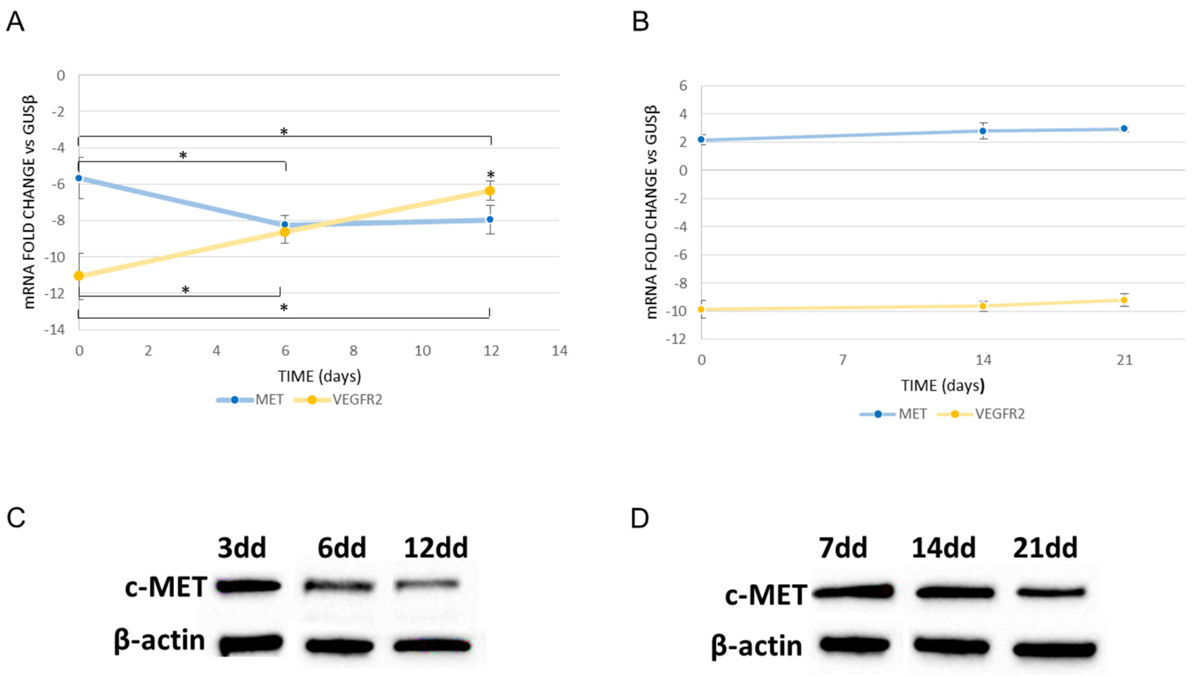

Supplementary Figure 1: c-MET and VEGFR2 gene and protein expression levels detected during osteoclast A e C. and osteoblast B e D. differentiation. *($p < 0.05$).

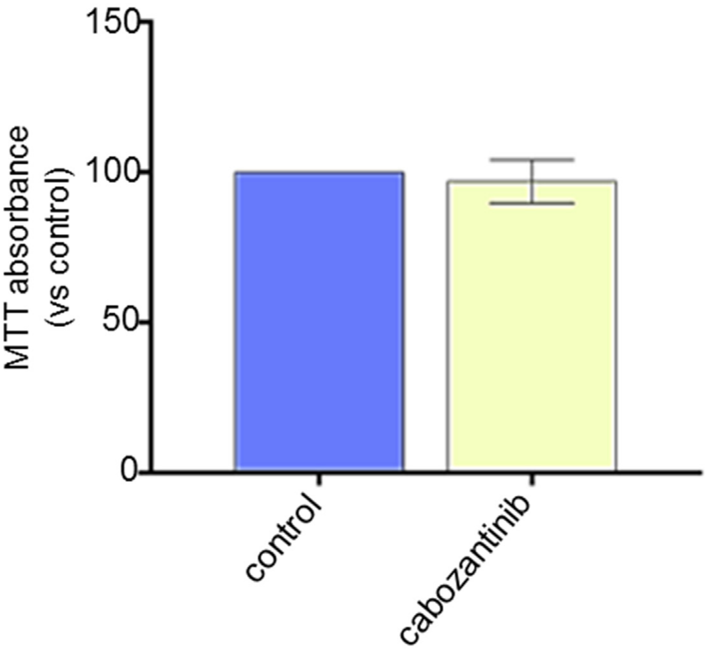

Supplementary Figure 2: Effect of cabozantinib treatment on osteoclast viability (MTT assay).

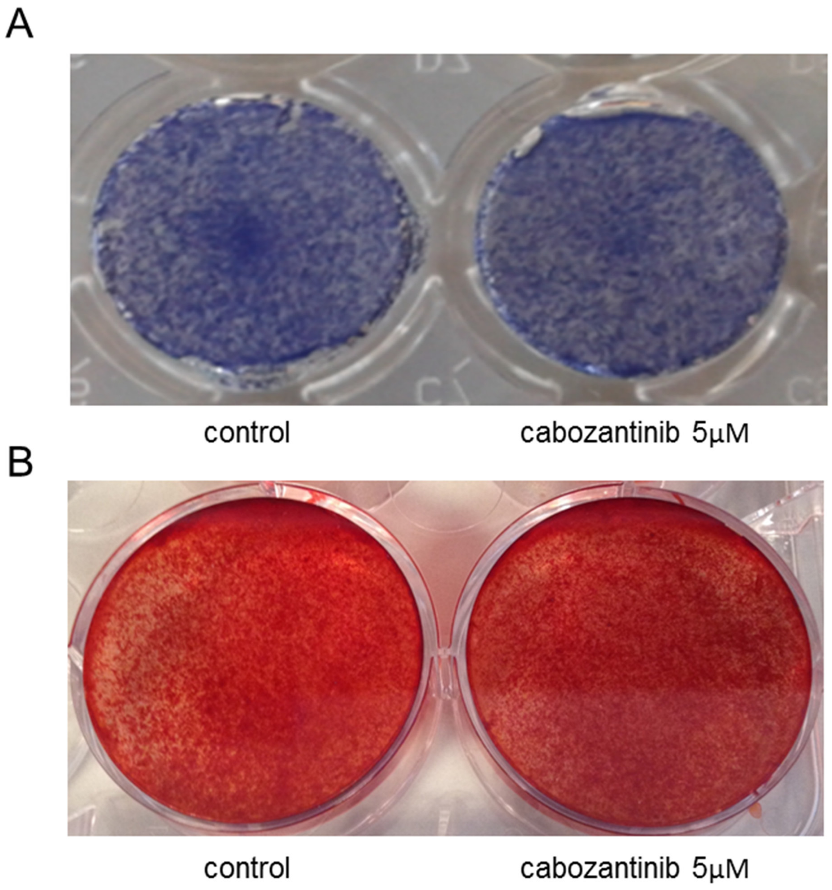

Supplementary Figure 3: Effect of cabozantinib treatment on osteoblast differentiation and activity. Representative images of ALP **A.** and Alizarin Red assay **B.** in untreated (DMSO) and treated osteoblasts.
